# Mental Health Outcomes Among Healthcare Workers and the General Population During the COVID-19 in Italy

**DOI:** 10.3389/fpsyg.2020.608986

**Published:** 2020-12-08

**Authors:** Rodolfo Rossi, Valentina Socci, Francesca Pacitti, Sonia Mensi, Antinisca Di Marco, Alberto Siracusano, Giorgio Di Lorenzo

**Affiliations:** ^1^Chair of Psychiatry, Department of Systems Medicine, University of Rome Tor Vergata, Rome, Italy; ^2^Department of Biotechnological and Applied Clinical Sciences, University of L’Aquila, L’Aquila, Italy; ^3^Department of Anesthesiology, Intensive Care and Emergency Medicine, IRCCS Fondazione Policlinico Universitario A. Gemelli, Rome, Italy; ^4^Department of Information Engineering, Computer Science and Mathematics, University of L’Aquila, L’Aquila, Italy; ^5^Psychiatry and Clinical Psychology Unit, Fondazione Policlinico Tor Vergata, Rome, Italy; ^6^IRCCS Fondazione Santa Lucia, Rome, Italy

**Keywords:** depression, anxiety, epidemiology, PTSD, risk factors

## Abstract

**Introduction:**

During the COVID-19 pandemic, healthcare workers in Italy have been exposed to an unprecedented pressure and traumatic events. However, no direct comparison with the general population is available so far. The aim of this study is to detail mental health outcomes in healthcare workers compared to the general population.

**Methods:**

24050 respondents completed an on-line questionnaire during the contagion peak, 21342 general population, 1295 second-line healthcare workers, and 1411 front-line healthcare workers. Depressive, anxious, post-traumatic symptoms and insomnia were assessed. Specific COVID-19 related potential risk factors were also considered in healthcare workers.

**Results:**

Depressive symptoms were more frequent in the general population (28.12%) and front-line healthcare workers (28.35%) compared to the second-line healthcare workers (19.98%) groups. Anxiety symptoms showed a prevalence of 21.25% in the general population, 18.05% for second-line healthcare workers, and 20.55% for front-line healthcare workers. Insomnia showed a prevalence of 7.82, 6.58, and 9.92% for the general population, second-line healthcare workers, and front-line healthcare workers, respectively. Compared to the general population, front-line healthcare workers had higher odds of endorsing total trauma-related symptoms. Both second-line healthcare workers and front-line healthcare workers had higher odds of endorsing core post-traumatic symptoms compared to the general population, while second-line healthcare workers had lower odds of endorsing negative affect and dissociative symptoms. Higher total traumatic symptom score was associated with being a front-line healthcare worker, having a colleague infected, hospitalized, or deceased, being a nurse, female gender, and younger age.

**Conclusion:**

This study suggests a significant psychological impact of the COVID-19 pandemic on the Italian general population and healthcare workers. Front-line healthcare workers represent a specific at-risk population for post-traumatic symptoms. These findings underline the importance of monitoring and intervention strategies.

## Introduction

Beginning in late February 2020 Italy has been the first European country to face the COVID-19 pandemic. Despite evidence of a relevant impact of the lockdown measures on mental health in the general population (GP) (Rossi et al., 2020b), healthcare workers (HCW) were exposed to a number of additional stressful events while working under extreme pressure with COVID-19 patients, and thus represent a highly at-risk population (Rossi et al., 2020a).

Challenges for staff include the increased workload and physical exhaustion due to the severe condition of the patients, witnessing a higher-than-usual death’s rate among their patients, fears of contagion for themselves and their families and seeing colleagues falling ill or dying (Walton et al., 2020; Zhang Y. et al., 2020).

Indeed, in the very early stages of the pandemic, the Italian national healthcare service and its regional articulations were subject to a never seen before pressure, with most intensive care units (ICU) running short of beds in a few days. Furthermore, lack of preparation for such a pandemic resulted in lack of security protocols and protection devices for HCW, which resulted in a tremendously high number of infected and deceased personnel.

Italian media stressed the war-like scenario in which ICU were working, allegedly performing triage with physician having to cherry-pick which patient to provide care to.

The psychological impact of this unprecedented health emergency might have significant long-term reverberations. Also, addressing the exact consequences of the COVID-19 pandemic on mental health of HCW is additionally critical (Firew et al., 2020), as mental health issues may hinder working ability of medical staff. For this reason, supportive interventions for HCW are necessary at this stage.

Despite the huge number of publications on the mental health burden in HCW, very few data have been published so far.

Recent reviews and original investigations confirm a high rate of anxious and depressive symptoms, as well as poor sleep quality and post-traumatic symptoms (Johnson et al., 2020), among HCW (Chew et al., 2020; Pappa et al., 2020; Talevi et al., 2020; Vindegaard and Benros, 2020).

Preliminary data from China during the COVID-19 pandemic showed a depression rate 50.3%, anxiety 44.6%, and insomnia 34.0% (Lai et al., 2020), although another works from China report lower rates of anxiety and depression in medical HCW (Huang and Zhao, 2020; Zhang W. et al., 2020). Kang et al. (2020) found that as much as 36% of medical staff reported subthreshold psychological symptoms.

In this scenario, we reported preliminary data on the very immediate burden of the COVID-19 outbreak on mental health on 1300 Italian HCW, finding that frontline young women, regardless of the working position (i.e., nurse, physician, healthcare assistant (HCA), etc.), had higher odds of several mental health outcomes, including PTSD symptoms, anxiety, depression and insomnia (Rossi et al., 2020a). We identified a number of job-related risk factors, including having a colleague infected, hospitalized, or deceased by COVID-19. Working directly with COVID-19 patients, i.e., being a Frontline HCW, was a major risk factor for all of the selected outcomes.

However, our preliminary data left some unsolved questions. Firstly, the odds of negative mental health outcomes in HCW compared to GP remains to be elucidated. Secondly, considering potential differences in the degree of exposure to a number of COVID-19 related traumatic events in HCW and GP, a more in-depth analysis of post-traumatic symptoms warrants further consideration.

### Aim of the Study

In this article, we aim to further detail mental health outcomes in an enlarged sample of HCW, with particular focus on post-traumatic symptoms (PTSS), and to compare selected outcomes between HCW and GP. Further, COVID-19 related risk factors were selected in order to capture potentially stressful events related to the increased pressure on the workplace.

## Materials and Methods

### Study Design

This cross-sectional web-based observational study is a part of a long-term monitoring program of mental health outcomes in the general population and health care workers. On-line consent was obtained from the participants, that were allowed to terminate the survey at any time they desired. The survey was anonymous, and confidentiality of information was assured. Three weeks after the beginning of the lockdown, a survey was conducted among a self-selected sample. Every person living in Italy ≥18 years was eligible. Approval for this study was obtained from IRB at the University of L’Aquila. This study adheres to the Declaration of Helsinki.

### Sampling Strategy and On-Line Questionnaire Dissemination

For the purpose of this study, two versions of an online questionnaire, one for the general population and one for HCW, were spread across the Italian population between March 25th and April 7th. The two questionnaires included the same psychometric measures but differed in the risk factors explored (see below). The general population questionnaire was spread using sponsored adverts on Facebook, while the HCW was spread using targeted posts on thematic Facebook groups and pages, as well as using a snowball spreading technique starting from the researchers’ acquaintances. Both versions of the questionnaire asked the participants to re-share the questionnaire link. Finally, the general population questionnaire included a link to the HCW version on its first page: in case an HCW encountered the GP link on-line, he/she was prompted to move to the HCW version of the questionnaire. Because of the particular dissemination technique, it was not possible to have precise data on response rate, however, using the Facebook Ads app, it was possible to estimate that the number of link clicks was about 100.000, while nearly one million people were reached by the ad.

### Outcome Measures

The time frame for all of the following psychometric instruments was set to the last 2 weeks.

The Italian version Global Psychotrauma Screen (GPS) (Olff et al., 2020; Rossi et al., 2020c) is a 22 self-report instrument with yes/no answers that covers both stress-related symptoms and risk and protective factors. Symptoms investigated are (17 items): post-traumatic stress symptoms, depression, sleep problems, dissociation, dysfunctional coping strategies including substance abuse and self-harm, and other physical, emotional, or social problems. Risk and protective factors are (5 items): other stressful events, childhood trauma, history of mental illness, social support, and psychological resilience.

The following scores were derived from the GPS.

(1) “GPS symptoms” (GPS-Sym): this score is the sum of all 17 symptoms items. Internal consistency α = 0.81.
(2) “GPS-post-traumatic symptoms” (GPS-PTSS): this score aggregates 4 items including core post-traumatic symptoms, i.e., re-experiencing, avoidance, hyperarousal, and insomnia. Internal consistency α = 0.63.
(3) “GPS-Negative affective symptoms” (GPS-NegAff): this score evaluates 11 items including symptoms related to disturbances in self-organization (DSO), anxiety, depression, self-harm, substance abuse, and other physical, emotional, or social problems. This cluster of symptoms qualifies complex post-traumatic symptoms and it is related to complex PTSD. Internal consistency α = 0.76.
(4) “GPS-dissociative symptoms” (GPS-Diss): this score includes depersonalization and derealization. Internal consistency α = 0.41.

In order to address COVID-related post-traumatic symptoms, items 1 and 2, regarding re-experiencing and avoidance, respectively, were slightly rephrased, referring to COVID-specific events or situations.

The Italian version of the 9-item Patient Health Questionnaire (PHQ-9) was used to assess depression. PHQ-9 comprises nine depressive symptoms, rated on a 4-point Likert scale, range 0–27). The total score has been taken into consideration as a continuous variable. PHQ-9 is a widely used instrument in epidemiological research as a depression screener. In our sample, internal consistency was *a* = 0.87.

The Italian version of the 7-item Generalized Anxiety Disorder questionnaire (GAD-7) was used to assess anxiety symptoms. GAD-7 includes 7 symptoms, rated on a 4-point Likert scale, range 0–21 (Spitzer et al., 2006). The total score has been taken into consideration as a continuous variable. GAD-7 is a widely used instrument in epidemiological research as an anxiety screener. In our sample, internal consistency was *a* = 0.91.

The Italian version of the 7-item Insomnia Severity Index (ISI) was used to assess sleep problems. ISI is a 7-item self-report questionnaire assessing the nature, severity, and impact of insomnia, on a 5-point Likert scale, range 0–28, with higher scores indicating higher severity of insomnia symptoms (Bastien et al., 2001; Castronovo et al., 2016). The total score has been taken into consideration as a continuous variable. ISI is a widely used instrument to evaluate sleep disorders. In our sample, internal consistency was *a* = 0.90.

### Exposure Measures, Covariates, and Confounders

The following COVID-related potential stressful exposures were assessed in the HCW cohort:

• working in direct contact with COVID-19 patients (i.e., front-line vs. second-line HCW);
• being exposed, infected, or hospitalized due to COVID-19;
• having a colleague who was infected, hospitalized, or deceased due to COVID-19;
• having been re-assigned to a different unit;
• job: Physician, Nurse, Healthcare Assistant, Other HCW (includes technicians, lab staff, and other health care workers).

The following potential confounders were selected in the two cohort:

• gender;
• age;
• geographical Area (Northern Italy: Aosta Valley, Piedmont, Lombardy, Liguria, Trentino-Alto Adige, Veneto, Friuli-Venezia Giulia, Emilia-Romagna; Center Italy: Tuscany, Umbria, Marche, Lazio; Southern Italy: Abruzzo, Molise, Puglia, Campania, Calabria, Basilicata, Sicily and Sardinia);
• education level: lower education, undergraduate, graduate, post-graduate degree.

### Statistical Analysis

Descriptive analyses were performed in order to assess the rates of mental health outcomes in the sample as well as the prevalence of the selected risk factors.

A panel of logistic or linear regression analyses was conducted – as appropriate depending on the dependent variable being continuous or binomial, in order to assess the association between risk factors and outcomes. Firstly, the association between belongingness to one of the three groups was explored as a putative risk factor. Selected confounders were introduced in subsequent analysis. Secondly, HCW-specific risk factors were tested in the HCW group.

## Results

### Sample Characteristics

Sample characteristics as well as rates of mental health outcomes are reported in Table 1. A total of 24050 respondents completed the questionnaire, of which 21342 were general population respondents (GP), 1295 were second-line healthcare workers (SHCW) and 1411 were front-line healthcare workers (FHCW).

**TABLE 1:** Sample characteristics.

	General Population (GP)	Second-line Health Care Workers (SHCW)	Frontline Health Care Workers (FHCW)
***N***	21342	1295	1411
**Gender (Female)**	17,183 (80.52%)	1,025 (79.15%)	1,125 (79.73%)
**Age**	38.95 (12.77)	43.47 (11.2)	40.64 (10.28)
**Region**			
*North*	9500 (45.21%)	506 (40.51%)	932 (67.83%)
*Center*	5325 (25.34%)	416 (33.31%)	306 (22.27%)
*South*	6188 (29.45%)	327 (26.18%)	136 (9.9%)
**Job**			
*Homemaker*	1481 (6.94%)	–	–
*Unemployed*	2586 (12.12%)	–	–
*Employed*	13006 (60.94%)	–	–
*Retired*	378 (1.77%)	–	–
*Student*	3891 (18.23%)	–	–
*Other HCW*	–	396 (30.58%)	300 (21.26%)
*Nurse*	–	397 (30.66%)	578 (40.96%)
*Physician*	–	302 (23.32%)	356 (25.23%)
*Gp*	–	42 (3.24%)	42 (2.98%)
*Non-specialist Physic*	–	20 (1.54%)	20 (1.42%)
*Healthcare Assistant*	–	138 (10.66%)	115 (8.15%)
**Education**			
*Lower education*	2043 (9.57%)	38 (2.93%)	41 (2.91%)
*High School*	10238 (47.9%)	249 (19.23%)	335 (23.74%)
*Graduate*	6572 (30.79%)	527 (40.6%)	533 (37.77%)
*Post-Graduate*	2489 (11.66%)	472 (36.45%)	497 (35.22%)

In the total sample, 19334 (80.39%) were female, independently of the group ($\chi_{2}^{2}$ = 1.867, *p* = 0.393). Mean age was 39.3 years (range: 18 to 88; SD = 12.6), with GP having a lower mean age (38.95; SD = 12.77) compared to SHCW (43.47; SD = 11.2) and FHCW (40.6; SD = 10.28).

Geographical distribution showed a higher abundance of FHCW in the northern regions compared to central and southern Italy ($\chi_{4}^{2}$ = 364.543, *p* < 0.001).

### Prevalence of Mental Health Outcomes

Prevalence of mental health outcomes is reported in Table 2. Depressive symptoms (PHQ-9 ≥ 15) were more frequent in the GP (28.12%) and FHCW (28.35%) compared to the SHCW (19.98%) groups ($\chi_{2}^{2}$ = 40.551; *p* < 0.001). Anxiety symptoms (GAD ≥ 15) showed a more balanced distribution among the three groups, with a prevalence of 21.25% for the GP group, 18.05% for SHCW and 20.55% for FHCW ($\chi_{2}^{2}$ = 7.706; *p* = 0.021). Similarly, insomnia symptoms showed a prevalence of 7.82%, 6.58% and 9.92% for the GP, SHCW and FHCW group, respectively ($\chi_{2}^{2}$ = 11.209; *p* = 0.004).

**TABLE 2:** Psychopathology and prevalence of mental health outcomes.

					Bonferroni *post hoc* test		
	
	General Population (GP)	Second-line Health Care Workers (SHCW)	Frontline Health Care Workers (FHCW)	Statistics (ANOVA or χ^2^)	GP vs. SHCW	GP vs. FHCW	SHCW vs. FHCW
*PHQ Tot*	10.67 (6.39)	9.49 (5.67)	11.03 (5.76)	*F*_2,23979_ = 24.16; *p* < 0.001	*p* < 0.001	*p* = 0.122	*p* < 0.001
*PHQ* ≥ *15*	5984 (28.12%)	258 (19.98%)	400 (28.35%)	$\chi_{2}^{2}$ = 40.551; *p* < 0.001			
*GAD Tot*	9.03 (5.95)	8.54 (5.61)	9.54 (5.41)	*F*_2,23973_ = 9.62; *p* = 0.001	*p* = 0.013	*p* = 0.005	*p* < 0.001
*GAD* ≥ *15*	4520 (21.25%)	233 (18.05%)	290 (20.55%)	$\chi_{2}^{2}$ = 7.706; *p* = 0.021			
*ISI Tot*	10.42 (7.26)	10.26 (7.10)	11.68 (7.01)	*F*_2,23995_ = 20.65; *p* < 0.001	*p* = 1.00	*p* < 0.001	*p* < 0.001
*ISI* ≥ *22*	1665 (7.82%)	85 (6.58%)	140 (9.92%)	$\chi_{2}^{2}$ = 11.209; *p* = 0.004			
*GPS-sym*	7.22 (3.85)	6.78 (3.66)	7.88 (3.44)	*F*_2,24021_ = 29.23; *p* < 0.001	*p* < 0.001	*p* < 0.001	*p* < 0.001
*GPS-PTSS*	2.11 (1.36)	2.33 (1.36)	2.63 (1.2)	*F*_2,24028_ = 106.32; *p* < 0.001	*p* < 0.001	*p* < 0.001	*p* < 0.001
*GPS-NegAff*	4.53 (2.63)	3.96 (2.39)	4.67 (2.32)	*F*_2,24027_ = 31.71; *p* < 0.001	*p* < 0.001	*p* = 0.176	*p* < 0.001
*GPS-Diss*	0.57 (0.66)	0.47 (0.62)	0.59 (0.66)	*F*_2,24021_ = 14.43; *p* < 0.001	*p* < 0.001	*p* = 1.00	*p* < 0.001

Regarding GPS sub-scores, GPS-Sym was 7.22 (SD = 3.85) in the GP group, 6.78 (SD = 3.66) in the SHCW group and 7.88 (SD = 3.44) in the FHCW group (*F*_2,24021_ = 29.23; *p* < 0.001). GPS-PTSS score was 2.11 (SD = 1.36), 2.33 (SD = 1.36) and 2.63 (SD = 1.2) in the GP, SHCW and FHCW, respectively (*F*_2,24028_ = 106.32; *p* < 0.001). GPS-NegAff score was 4.53 (SD = 2.63) in the GP group, 3.96 (SD = 2.39) in the SHCW group and 4.67 (SD = 2.32) in FHCW group (*F*_2,24027_ = 31.71; *p* < 0.001). GPS-Diss score was 0.57 (SD = 0.66) in the GP group, 0.47 (SD = 0.62) in SHCW group and 0.59 (SD = 0.66) in the FHCW group (*F*_2,24021_ = 14.43; *p* < 0.001). Bonferroni *post hoc* test showed that all pairwise comparison were statistically significant, except for GP vs. FHCW on the GPS-NegAff and GPS-Diss subscale.

### Regression Analyses

Results from the first panel of regressions are reported in Table 3. Compared to the GP group, SHCW had lower odds of endorsing depressive, anxious, and lower levels of total trauma related symptoms (GPS-Sym), with higher levels of core PTSS (GPS-PTSS) and lower levels of trauma-related negative affective symptoms (GPS-NegAff).

**TABLE 3:** Logistic and linear regression of group category on mental health outcomes.

*Unadjusted*	*PHQ-9*		*GAD-7*		*ISI*		*GPS-Sym*		*GPS-PTSS*		*GPS-NegAff*		*GPS-Diss*	
							
	*OR [95% CI]*	*p*	*OR [95% CI]*	*p*	*OR [95% CI]*	*p*	*b [95% CI]*	*p*	*b [95% CI]*	*p*	*b [95% CI]*	*p*	*b [95% CI]*	*p*
**GP**	Ref													
**SHCW**	0.64*** [0.56,0.73]	< 0.001	0.82** [0.71,0.94]	0.0063	0.83 [0.66,1]	0.11	−0.44*** [−0.66, −0.23]	< 0.001	0.22*** [0.15,0.3]	< 0.001	−0.57*** [−0.72, −0.42]	< 0.001	−0.1*** [−0.14, −0.064]	< 0.001
**FHCW**	1 [0.9,1.1]	0.85	0.96 [0.84,1.1]	0.54	1.3** [1.1,1.6]	0.0048	0.65***[0.45, 0.86]	< 0.001	0.51*** [0.44, 0.59]	< 0.001	0.14 [−0.01, 0.27]	0.059	0.01 [−0.03, 0.04]	0.641
***Adjusted***^§^														
**GP**	Ref.													
**SHCW**	0.68*** [0.58,0.78]	< 0.001	0.89 [0.76,1]	0.124	0.73** [0.57,0.93]	0.01	−0.19 [−0.4,0.029]	0.089	0.25*** [0.17,0.33]	< 0.001	−0.35*** [−0.5, −0.21]	< 0.001	−0.09*** [−0.12, −0.047]	< 0.001
**FHCW**	1 [0.91,1.2]	0.651	0.98 [0.85,1.1]	0.786	1.2* [1,1.5]	0.025	0.73*** [0.52,0.94]	< 0.001	0.55*** [0.47,0.62]	< 0.001	0.17* [0.029,0.31]	0.018	0.017 [−0.021,0.054]	0.384

GP, general population; SHCW, second-line healthcare workers; FHCW, frontline health care workers; PHQ-9, patient health questionnaire; GAD-7, generalized anxiety disorder questionnaire; ISI, insomnia severity index; GPS-Sym, global psychotrauma screen – total symptom score; PTSS, post-traumatic symptoms; NegAff, negative affective symptoms; Diss, dissociative symptoms.§adjusted by age gender region education. *p < 0.05; **p < 0.01; ***p < 0.001. Effect of the independent variable on PHQ, GAD and ISI were estimated using logistic regression; effects of the independent variable on GPS total score and subscores were estimated using linear regression.

FHCW had higher odds of endorsing insomnia and trauma-related symptoms (GPS-Sym). Regarding GPS sub-scores, both SHCW and FHCW had higher odds of endorsing core PTSS (GPS-PTSS score) compared to the GP group, while SHCW had lower odds of endorsing symptoms from the negative affect and dissociative cluster (GPS-NegAff and GPS-Diss scores).

**FIGURE 1:**
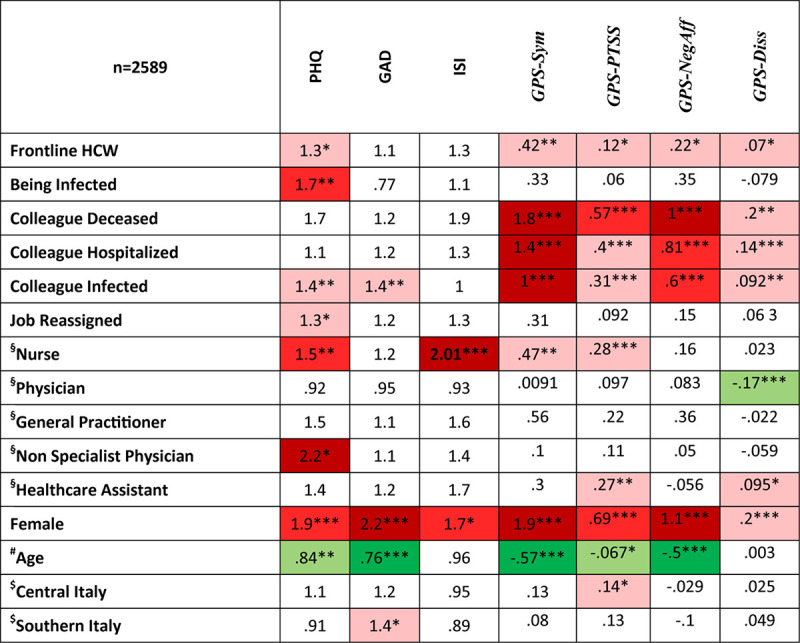
Summary of risk factors for mental health outcomes in healthcare workers. Numbers in cells represent odds ratios for PHQ, GAD, and ISI and linear regression coefficients for GPS subscales. Red highlight: statistically significant positive association between risk factor and outcome. Green highlight: statistically significant negative association between risk factor and outcome. PHQ, patient health questionnaire; GAD, generalized anxiety disorder questionnaire; ISI, insomnia severity index; GPS, global psychotrauma screen; GPS-Sym, GPS total score; GPS-PTSS, GPS post-traumatic Symptoms; GPS-NegAff, GPS negative affect; GPS-Diss, GPS dissociative symptoms. §Reference category: other health care worker; #Standardized age. Negative associations show that younger age is associated with worst outcomes. $Reference category: Northern Italy; *p < 0.05; **p < 0.01; ***p < 0.001.

Concerning putative risk factors (Table 4 and Figure 1), for HCW, depressive symptoms were associated with being an FHCW, being infected by COVID-19, having a colleague infected, being reassigned to a different job, being a nurse or a non-specialist physician, female gender and younger age. Anxious symptoms were associated with being infected, female gender, and younger age. Insomnia was associated with being a nurse and a female gender. A higher total GPS symptom score was associated with being an FHCW, having a colleague infected, hospitalized, or deceased, being a nurse, female gender, and younger age. Of the GPS subscales, PTSS were associated with being an FHCW, having a colleague infected, hospitalized or deceased, being a nurse or a Healthcare Assistant (HCA), female gender, and younger age.

**TABLE 4:** Logistic and linear regression of potential risk factors on mental health outcomes in healthcare workers.

*n* = 2589	PHQ		GAD		ISI		*GPS-Sym*		*GPS-PTSS*		*GPS-NegAff*		*GPS-Diss*	
							
	*OR [95% CI]*	*p*	*OR [95% CI]*	*p*	*OR [95% CI]*	*p*	*b [95% CI]*	*p*	*b [95% CI]*	*p*	*b [95% CI]*	*p*	*b [95% CI]*	*p*
**Frontline**	1.3* [1,1.6]	0.022	1.1 [0.84,1.3]	0.64	1.3 [0.94,1.8]	0.1	0.42** [0.12,0.72]	0.006	0.12* [0.012,0.23]	0.031	0.22* [0.025,0.42]	0.028	0.07* [0.014,0.13]	0.014
**Infected**	1.7** [1.2,2.4]	0.004	0.77 [0.48,1.2]	0.26	1.1 [0.65,2]	0.67	0.33 [−0.25,0.91]	0.261	0.06 [−0.16,0.28]	0.588	0.35 [−0.035,0.74]	0.075	−0.079 [−0.19,0.03]	0.157
**Colleagues involved**														
***Colleagues not involved***	Ref.													
***Deceased***	1.7 [1,2.8]	0.05	1.2 [0.67,2.2]	0.52	1.9 [0.97,3.9]	0.062	1.8*** [1,2.6]	< 0.001	0.57*** [0.29,0.86]	< 0.001	1*** [0.51,1.5]	< 0.001	0.2** [0.06,0.35]	0.006
***Hospitalized***	1.1 [0.88,1.5]	0.31	1.2 [0.87,1.6]	0.31	1.3 [0.85,1.9]	0.24	1.4*** [0.98,1.7]	< 0.001	0.4*** [0.26,0.54]	< 0.001	0.81*** [0.57,1.1]	< 0.001	0.14*** [0.071,0.21]	< 0.001
***Infected***	1.4** [1.1,1.8]	0.002	1.4** [1.1,1.8]	0.007	1 [0.71,1.5]	0.92	1*** [0.68,1.3]	< 0.001	0.31*** [0.19,0.43]	< 0.001	0.6*** [0.38,0.81]	< 0.001	0.092** [0.031,0.15]	0.003
**Job reassigned**	1.3* [1,1.7]	0.021	1.2 [0.94,1.6]	0.12	1.3 [0.87,1.8]	0.22	0.31 [−0.071,0.68]	0.112	0.092 [−0.049,0.23]	0.202	0.15 [−0.097,0.41]	0.229	0.06 [−0.011,0.13]	0.095
**Job**														
***Other HCW***	ref													
***Nurse***	1.5** [1.2,1.9]	0.001	1.2 [0.92,1.6]	0.18	2.01*** [1.3,3]	< 0.001	0.47** [0.12,0.82]	0.008	0.28*** [0.15,0.41]	< 0.001	0.16 [−0.067,0.4]	0.165	0.023 [−0.042,0.088]	0.483
***Physician***	0.92 [0.69,1.2]	0.56	0.95 [0.71,1.3]	0.75	0.93 [0.57,1.5]	0.78	0.0091 [−0.37,0.39]	0.963	0.097 [−0.046,0.24]	0.183	0.083 [−0.17,0.34]	0.522	−0.17*** [−0.24, −0.1]	< 0.001
***Gp***	1.5 [0.81,2.6]	0.21	1.1 [0.56,2.1]	0.82	1.6 [0.66,3.8]	0.31	0.56 [−0.25,1.4]	0.177	0.22 [−0.082,0.53]	0.153	0.36 [−0.18,0.9]	0.191	−0.022 [−0.17,0.13]	0.777
***Non-specialist phy*∼*n***	2.2* [1.1,4.4]	0.026	1.1 [0.5,2.5]	0.78	1.4 [0.41,4.9]	0.57	0.1 [−1,1.2]	0.852	0.11 [−0.3,0.53]	0.588	0.05 [−0.68,0.78]	0.895	−0.059 [−0.27,0.15]	0.578
***HCA***	1.4 [0.98,2]	0.062	1.2 [0.82,1.8]	0.34	1.7 [0.99,3]	0.053	0.3 [−0.2,0.81]	0.235	0.27** [0.078,0.45]	0.006	−0.056 [−0.39,0.28]	0.742	0.095* [0.00064,0.19]	0.048
**Male**	Ref													
**Female**	1.9*** [1.5,2.5]	< 0.001	2.2*** [1.7,3]	< 0.001	1.7* [1.1,2.6]	0.014	1.9*** [1.6,2.3]	< 0.001	0.69*** [0.56,0.81]	< 0.001	1.1*** [0.84,1.3]	< 0.001	0.2*** [0.14,0.26]	< 0.001
**Age**^§^	0.84** [0.75,0.94]	0.002	0.76*** [0.67,0.86]	< 0.001	0.96 [0.81,1.1]	0.64	−0.57*** [−0.72, −0.41]	< 0.001	−0.067* [−0.13, −0.01]	0.025	−0.5*** [−0.61, −0.4]	< 0.001	0.003 [−0.026,0.033]	0.817
**Region**														
***North***	Ref													
***Center***	1.1 [0.89,1.4]	0.33	1.2 [0.95,1.5]	0.13	0.95 [0.67,1.4]	0.79	0.13 [−0.19,0.45]	0.412	0.14* [0.019,0.26]	0.023	−0.029 [−0.24,0.18]	0.786	0.025 [−0.035,0.085]	0.411
***South***	0.91 [0.68,1.2]	0.51	1.4* [1,1.8]	0.033	0.89 [0.56,1.4]	0.6	0.08 [−0.31,0.47]	0.686	0.13 [−0.01,0.28]	0.068	−0.1 [−0.36,0.15]	0.431	0.049 [−0.024,0.12]	0.186

GP, general population; SHCW, second-line healthcare workers; FHCW, frontline health care workers; PHQ-9, patient health questionnaire; GAD-7, generalized anxiety disorder questionnaire; ISI, insomnia severity index; GPS-Sym, global psychotrauma screen – total symptom score; PTSS, post-traumatic symptoms; NegAff, negative affective symptoms; Diss, dissociative symptoms; HCW, health care worker; HCA, health care assistant. §adjusted by age gender region education. *p < 0.05; **p < 0.01; ***p < 0.001. Effect of the independent variable on PHQ, GAD, and ISI were estimated using logistic regression; effects of the independent variable on GPS total score and subscores were estimated using linear regression.

## Discussion

In this article, we report on the mental health outcomes of a sample of Italian HCW and a GP sample during the peak of the critical infection of the COVID-19 outbreak. Preliminary data from a part of this sample were previously published elsewhere (Rossi et al., 2020a,b). Results confirmed high rates of depression symptoms, anxiety symptoms, insomnia, and PTSS both in the GP and HCW group during the acute phase of the COVID-19 emergency. Further, we aimed at addressing, for the first time, mental health outcomes in front and second-line HCW and the GP together, allowing a comparison among the three groups.

In this respect, we firstly compared mental health outcomes among FHCW, SHCW, and the GP groups, finding that being an SHCW was associated with lower odds of endorsing anxious or depressive symptoms compared to the GP, while being a front and second-line HCW was associated with higher PTSS compared to the general population. GPS-Negative Affect symptoms were associated with being FHCW compared to the GP in the adjusted model.

Our data suggest a complex pattern of distribution of mental health outcomes among the three groups.

SHCW have a more positive outcome profile compared to both GP and FHCW, except for PTSS. This result was kept after controlling for education level, age, and gender. In order to explain this result, based on previous evidence (Gan et al., 2020; Rossi et al., 2020b), we assume that much of the impact on depressive and anxious symptoms in the GP could be due to lockdown measures, social distancing, and economic instability in addition to traumatic experiences. SHCW could have been less exposed to lockdown measures, but not to social distancing, compared to the GP because they continued to work without being put under excessive pressure, and this could be a reason for which they were somewhat protected against depressive symptoms compared to the GP. On the other hand, SHCW may have been exposed to traumatic events at their workplace (especially indirect traumatic events, such as knowing of colleagues infected or deceased), hence showing an increase in PTSS symptoms compared to the GP.

However, this interpretation should be taken with caution, as the pattern of exposure to traumatic events, lockdown measures, and social distancing was not actually captured by our or others’ data, rather it relies on lay evidence of how HCW and GP working pattern changed during the assessed time period.

FHCW didn’t show an increase in depressive or anxious symptoms compared to the GP, while they showed a relevant increase in trauma-related symptoms. Furthermore, compared to SHCW, they showed higher levels of negative affective post-traumatic symptoms, suggesting that working as a front-line HCW is associated with a complex pattern of traumatic exposure, that could include physical and mental exhaustion, witnessing a high number of deaths of patients and colleagues and fear of contagion.

Secondly, we explored COVID-related risk factors for mental health outcomes in the HCW sub-sample only, finding that being an FHCW was associated with higher odds of endorsing depressive and post-traumatic symptoms compared to SHCW. The COVID-related risk factors explored were specifically associated with trauma-related symptoms such as PTSS and negative affect symptoms. In particular, colleagues’ negative events, i.e., being infected, hospitalized, or deceased, were all associated with PTSS and trauma-related negative affective symptoms.

Taken together, these results suggest that, although the COVID-19 pandemic has had a relevant impact on the general population’s mental health as a whole (Rossi et al., 2020b), HCWs are a population at heightened risk specifically for trauma-related symptoms.

Regarding putative risk factors in the HCW group, contrary to early data from Chinese HCW (Lai et al., 2020), in our sample, no specific working position was associated with higher odds of mental health outcomes, except for nurses and healthcare assistants having higher odds of insomnia. However, in line with previous data on both the GP and HCW (Liu et al., 2020; Qiu et al., 2020; Wang et al., 2020), being female was associated with all the mental health outcomes considered, suggesting that female gender represents a risk factor for mental health issues such as PTSS, Depression, Anxiety symptoms, and Insomnia in the context of the current emergency. Also, similarly to previous reports (Qiu et al., 2020; Wang et al., 2020), younger age was associated with all the selected mental health variables, except for Insomnia. These findings therefore further encourage the implementation of targeted interventions for different at-risk populations.

This study has a number of limitations, mainly due to the on-line sampling strategy and cross-sectional design. Firstly, a self-selection bias, which is frequent in web-based surveys, could have led to an overestimation of effect sizes. Moreover, it is possible that this effect was different in the HCW and GP subsamples, leading to a biased estimate of the group effect on the selected outcomes. Secondly, it was not possible to assess how many subjects were reached by the questionnaire, so a response rate could not be estimated. A different sampling strategy, based on mailing lists of medical associations could have yielded a more accurate sample, however, getting access to mailing lists owned by Local Health Authorities could have introduced a relevant delay in sampling, eventually causing us to miss the relevant timeframe for this study.

Thirdly, this study is based on self-report measures that inherently convey a systematic bias in estimated the target construct.

Lastly, this study is based on a cross-sectional design. Although follow-up data will be collected in the future, no baseline data on the same participants were available at the time of the recruitment, and the only epidemiological study available in Italy so far (Girolamo et al., 2006) dates back to 2006 and is based on very different data collection instruments, hampering any possible comparison with our data.

However, this study has several strengths as well, consisting in its large sample size, and the prompt data collection, that was conducted during the highest peak of contagions of COVID-19 and burden on the national health service.

### Clinical Implications

This study suggests a significant psychological impact of the COVID-19 pandemic on both the Italian GP and HCW. In this context, our results further underline the importance of timely intervention strategies, with particular regard to HCW. Indeed, specific attention should be dedicated to FHCW, a highly vulnerable population exposed to a number of additional emergency-related stressful events. Health care systems should cope with the psychological impact of the pandemic on HCW by actively monitoring mental health outcomes and performance, modifying working shifts, and reducing the exposure to frontline workplace HCW, especially those exposed to a higher risk of unfavorable mental health outcomes, such as trauma-related symptoms, should be provided with training, psychological support, and treatments where necessary. Early detection and intervention strategies in both the general population and at-risk groups are crucially important in order to prevent the potential long-term adverse psychological impact of large-scale emergencies such as the current COVID-19 pandemic. On the other hand, further studies should attempt to address any possible protective factors or positive coping styles that may have protected the population from the risk factors associated with the pandemic.

### Significant Outcomes

Front-line Health care workers are at heightened risk for Post-Traumatic symptoms. Second-line health care workers showed lower levels of depression and anxiety compared to the general population. Younger age and female gender, having a colleague involved with COVID-19 were associated with mental health outcomes.

### Limitations

On-line self-selected sample; self-report assessment.

## Data Availability Statement

The raw data supporting the conclusions of this article will be made available by the authors.

## Ethics Statement

The studies involving human participants were reviewed and approved by the University of L’Aquila IRB. The patients/participants provided their written informed consent to participate in this study.

## Author Contributions

RR, VS, FP, and GDL: conceptualization. RR: methodology and formal analysis. RR, SM, and GDL: data curation. RR and VS: writing–original draft. RR, VS, AD, FP, SM, AS, and GDL: writing–review and editing. All authors contributed to the article and approved the submitted version.

## Conflict of Interest

The authors declare that the research was conducted in the absence of any commercial or financial relationships that could be construed as a potential conflict of interest.
